# Variability Between Datasets and Statistical Approaches—Rethinking Estimation of Default Dermal Absorption Values for Risk Assessment

**DOI:** 10.3390/toxics13110925

**Published:** 2025-10-29

**Authors:** Veronika Städele, Sabine Martin, Korinna Wend

**Affiliations:** Department Pesticides Safety, German Federal Institute for Risk Assessment, Max-Dohrn-Straße 8-10, 10589 Berlin, Germany

**Keywords:** prediction interval, in vitro, guidance, active substance, exposure, PPP, Bayesian, linear model, *MCMCglmm*

## Abstract

In risk assessment, deriving dermal absorption values is essential for evaluating plant protection products. Applicants submit study data, which authorities assess during the authorisation process. If no data are provided, default values from the European Food Safety Authority 2017 Guidance on dermal absorption (EFSA GD2017) apply. The German Federal Institute for Risk Assessment compiled an updated dermal absorption dataset of 356 more recent human in vitro studies evaluated under to the newest guidance. We applied the same empirical and modelling approaches used to derive default values for concentrates (commercially available product concentrations) and dilutions in different formulation type categories in EFSA GD2017 to the new dataset and compared the resulting values. We also assessed the impact of applying the alternative definition of ‘concentrate’ (>50 g/L) according to SCoPAFF. Default values obtained by analysing the new dataset were considerably lower than current default values, particularly for solids applied in dilutions. The alternative definition of ‘concentrate’ did not have a large impact on default values. Our results suggest that a revision of the default values based on newer studies evaluated under the most current guidance may be warranted.

## 1. Introduction

Dermal absorption of active substances in their representative formulations is an integral part of the risk assessment of plant protection products (PPPs) in the European Union [1,2,3]. Absorption values are used to perform exposure estimates by translating dermal exposure, the primary route of exposure during application of PPPs [4], into potential systemic exposure. Persons who may come intentionally or unintentionally into contact with PPPs are operators, workers, bystanders, or residents. In vitro or in vivo dermal absorption studies can be submitted by an applicant and are subsequently evaluated by the competent authorities during the authorisation procedure [5,6]. Several guidance documents [4,7,8,9] are available to assist with critical aspects related to the design of dermal absorption studies and their evaluation. To explore a range of possible exposure scenarios, and because dermal absorption of active substances is usually concentration dependent in a non-linear fashion—at higher concentrations smaller fractions are absorbed—dermal absorption studies often test the commercially available concentrated product, as well as one or more intended spray dilutions [9,10,11,12,13,14].

In case no dermal absorption studies are provided by an applicant, default values may be used. Default values need to be easily implementable in risk assessment, be as realistic as possible to avoid non-approval of substances that could be safely used, but at the same time conservative enough to be protective to human health. Default values established in the European Food Safety Authority (EFSA) guidance document from 2017 (EFSA GD2017) [9] apply for risk assessment in the European Union. Values are expressed in percentage of active substance absorbed. Separate values are given for concentrates and dilutions, as well as for two groupings of formulation type categories, with concentrates defined as the concentration of active substance in the commercially available undiluted product: 10% for concentrates and 50% for dilutions in water-based or solid formulations; 25% for concentrates and 70% for dilutions in organic solvent or ‘other’ formulations [9]. These values were derived based on the outcome of two different statistical approaches to analysis (empirical and modelling) applied to a large dataset of dermal absorption experiments [9].

The existing default values might warrant reconsideration in light of potential improvements in the implementation of dermal absorption studies driven by technological advancements and their strict evaluation according to the newest guidance [13].

After publication of EFSA GD2017, the Standing Committee on Plants, Animals, Food, and Feed (SCoPAFF) published a corrigendum which redefined concentrates as concentrations of the active substances in the product of higher than 50 g/L (or 50 g/kg, 5%), and consequently defined dilutions as concentrations of the active substances in the product of equal to or lower than 50 g/L (or 50 g/kg, 5%) [15]. While most product concentrates fall above this threshold, some active substances may occur at much lower concentrations, particularly if more than one active substance is contained in a product. The impact of this redefinition of ‘concentrate’ and ‘dilution’ on default values has not yet been investigated.

In the course of its regulatory work, the German Federal Institute for Risk Assessment (BfR) has compiled an updated dataset of dermal absorption studies for PPPs—the BfR2024 dataset. This new dataset is based on studies submitted for the authorisation of PPPs and active substance evaluations. It has been compiled from study information entered in the publicly available EFSA dermal absorption template which is a harmonised template used in European Union regulation [16]. The dermal absorption study data include basic information such as target concentration and product formulation type, technical parameters such as diffusion cell type and exposure duration, as well as skin related parameters such as donor age and skin source. Absorption values for individual experimental replicates were extracted from the templates. The dataset includes studies evaluated up to 2024, whereas a prior BfR dataset included studies up to 2018 [13]. Studies were evaluated in accordance with the newest EFSA guidance on dermal absorption [9].

In this manuscript, we apply the same analyses used to derive the default values in EFSA GD2017 to the BfR2024 dataset. We compare the resulting values to the existing default values and to values obtained in Sarti et al. 2025 [10] to investigate the generalisability of the currently used default values with regard to other datasets, evaluate the impact of the redefinition of concentrates and dilutions, and discuss potential implications of the resulting differences.

## 2. Materials and Methods

### 2.1. Dermal Absorption Dataset (BfR2024)

The present dataset was compiled by computationally extracting data from filled-in harmonised EFSA templates for dermal absorption studies [16] which were manually populated by risk assessors using information provided in study reports. We then reviewed the data for missing entries and manually completed the dataset by consulting the original study reports. The data were subsequently checked for formatting and internal consistency. In the EFSA template, certain fields are populated using numerical entries or predefined options from drop-down menus, while others require free-text input. We further completed several plausibility checks on the numerical values, such as identification of general outliers, concentrates with extremely low concentrations, dilutions with extremely high concentrations, and whether target concentration, skin area, area dose and total dose were consistent and mathematically resulted in plausible application volumes and rates. We checked flagged entries manually against the original study reports.

**Figure 1:**
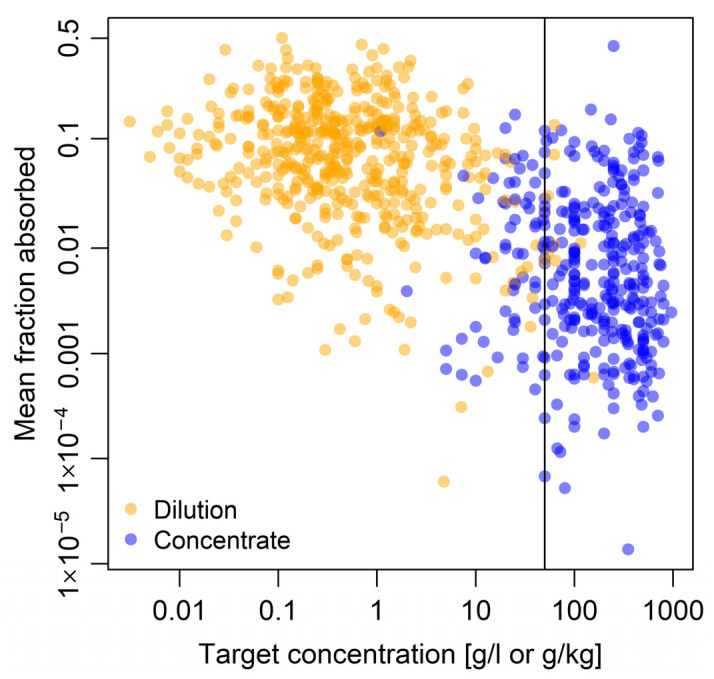
Relationship of target concentration and mean fraction absorbed. Mean fraction absorbed is the mean across replicates for dilutions and concentrates aggregated by substance and study. The *x*-axis is log-transformed (base 10) and the *y*-axis is logit-transformed. Concentrates represent the undiluted product; dilutions are intended in-use dilutions of a product. The vertical line shows the alternative classification of target concentrations into concentrates and dilutions using a cut-off at 50 g/L (g/kg) according to SCoPAFF [15].

The final dataset contained 6340 absorption values from 356 Good Laboratory Practice and Organisation for Economic Cooperation and Development (OECD)-compliant human in vitro dermal absorption studies on 155 active substances used in PPPs and one active substance metabolite in 20 different product formulation types (Table 1 and Table 2) [4,6,7,17]. Studies were conducted between 1999 and 2022. Most studies (92%, n = 328) investigated the absorption of only one substance, 7% of studies (n = 24) investigated two substances, and the remaining 1% of studies (n = 4) investigated three substances. Absorption of the active substance metabolite prothioconazole-desthio was investigated by the largest number of studies (6%, n = 20), absorption of glyphosate was examined by the second largest number of studies (4%, n = 14), but for most active substances (47%, n = 72), absorption was investigated in only one study. Target concentrations ranged from 0.003 to 960 g/L (or g/kg for solids) with a mean ± SD of 98 ± 173 g/L and a median of 2.5 g/L (Figure 1). The fraction absorbed ranged from 9 × 10^−6^ to 0.78 with a mean ± SD of 0.07 ± 0.10 and a median of 0.03 (Figure 1). In two thirds of studies (64%, n = 229), exposure of skin to active substances was 8 h and in about one third of studies (36%, n = 127) it was 6 h. As for analyses performed in EFSA GD2017 it was decided to apply the less stringent OECD [8] requirement for the mean mass-balance recovery, excluding experiments with mean recovery <90% and >110%. Absorption throughout this manuscript refers to potential absorption, i.e., active substance remaining in the skin at the end of sampling is included as it could potentially still be absorbed. Absorption was then calculated from the quantification of active substance in different compartments of the experimental setup as the material in the receptor fluid, the chamber wash, and the whole skin. In accordance with EFSA GD2017, if absorption was considered complete, i.e., at least 75% of the material was absorbed on average after half the sampling duration, all tape strips were excluded. If less than 75% of the material was absorbed on average after half the sampling duration, only tape strips 1 and 2 were excluded [9].

### 2.2. Statistical Analyses

Previous analyses of the influence of several factors (experimental, substance- and product-specific) on dermal absorption [9] revealed that the fraction absorbed was most strongly dependent on the type of product formulation and whether the product was applied as a concentrate or a dilution. For the main analysis, we defined concentrates as products in their commercially available concentration and dilutions as intended in-use dilutions of products. Following EFSA GD2017, we categorised product formulation types into four groups: solid, water-based, organic-solvent and other formulations (Table 1), and then split each group according to whether the product was applied as a concentrate or as a dilution, resulting in eight final categories (Table 3). Initial analyses in EFSA GD2017 were also conducted splitting the data into eight groups as described here. However, final default values are provided for only four groups, grouping the ‘organic solvent’ and ‘other’ categories, as well as the ‘water-based’ and ‘solid’ categories, as initial analyses found that each pair of categories produced similar default values when analysed separately. Detailed descriptions of formulation types can be found in [18].

To estimate the influence of the alternative definition of concentrates and dilutions according to SCoPAFF [15], we conducted a second set of analyses in which we defined concentrates as target concentrations of the active substance in the product higher than 50 g/L (g/kg), i.e., 5% active substance. Eight concentrations previously classified as dilutions were classified as concentrates under this definition, and 73 concentrations previously classified as concentrates were classified as dilutions (Figure 1). All analyses in this manuscript were conducted in R 4.4.1 [19].

**Figure 2:**
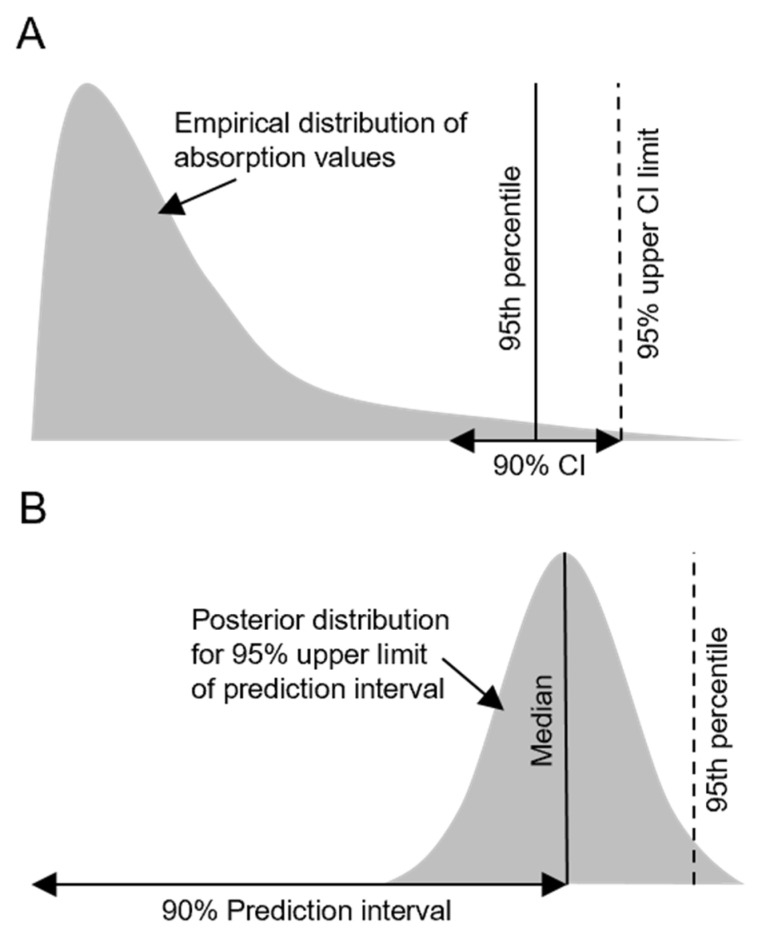
Schematic representation of how default values for dermal absorption were obtained in EFSA GD2017 [9] and this manuscript (**A**) with the empirical approach and (**B**) via Bayesian linear mixed modelling. 95th percentile: 95% of (empirical) values are lower than this value. 95% upper limit of prediction interval: 95% of future values are predicted to be lower than this value. Dashed lines represent the most conservative option for deriving default values. CI: Confidence interval.

To derive comparable default values for each category from the new dataset, we closely followed analyses performed in EFSA GD2017 which obtained default values in two ways: (1) as empirical percentiles of the observed distributions of absorption values, and (2) based on logit-regression modelling. Methodological details and code to perform the analyses are published [9], but we also provide a general description below. Default values need to be conservative estimates of absorption to be protective to human health. Both approaches account for this in different ways detailed in the following paragraphs and shown in Figure 2. The code and the data necessary to replicate the analyses are provided in the Supplementary Information.

#### 2.2.1. Default Values Based on Empirical Percentiles

For each category, we first calculated 95th percentiles of the distributions of absorption values. We obtained 95% upper confidence interval limits for these percentile values by determining the kth value in each empirical distribution of size n, where k is the number of successes in a binomial distribution of n draws (with a 95% probability of success for each draw) that would be observed with a cumulative probability of 95%. The upper confidence limit represents the most conservative empirical basis for deriving default values as it also takes into account uncertainty in the 95th percentile value. A schematic representation of the approach is shown in Figure 2A. The data violate the assumption of being a random sample from a homogeneous population, posited by the calculation of confidence limits in this way. Additionally, not accounting for substances and studies contributing multiple datapoints to the dataset limits the generalisability of the outcome. We include the approach to enable full comparison with EFSA GD2017 and refer to it as the ‘empirical approach’.

To account for clustering in the data as a source of heterogeneity, e.g., absorption differing among substances or studies, and for comparison with EFSA GD2017, we employed a second approach based on Bayesian linear mixed modelling.

#### 2.2.2. Default Values Based on Bayesian Mixed Models

The second approach aimed to account for the heterogeneity in the data by specifically incorporating some sources of heterogeneity in Bayesian linear mixed regression modelling. To this end, we constructed Gaussian linear mixed models using the *MCMCglmm* function in the *MCMCglmm* R-package version 2.36 [20]. The response variable was the logit-transformed fraction of substance absorbed, as prior investigations described in EFSA GD2017, and also replicated in this study, indicated that the assumptions of heteroscedasticity and normality would be best fulfilled under this transformation. Concentration status (concentrate vs. dilution) and formulation type category (solid, water-based, organic solvent, and other) were included as fixed effects with ‘dilution’ and ‘organic solvent’ as the respective reference categories. We included random effects for three variables: (1) a random slope for substance within concentration status, estimating between-substance variation for concentrates and dilutions separately, as well as their correlation; (2) a random intercept for study identity to account for between-study variation; (3) a random intercept for a nested synthetic variable for within-study variation which grouped replicates of the same applied concentration of the same substance in the same formulation type within the same study. The inclusion of random effects serves two main purposes in the context of this analysis. (1) Each random effect estimates how much each level of a random effect, for example each substance, differs from the average response. This means the analysis takes into account that datapoints contributed by the same substance are not independent, and that some substances contribute more datapoints than others, thereby allowing to estimate a generalisable average response [21]. This is a general advantage of employing so called mixed effect models which are preferred for unbalanced datasets. (2) It allows estimating variability among different levels of a random effect which can then be used to calculate prediction intervals as a basis for default values.

Hence, to derive default values, we obtained posterior distributions for the upper 95% credible limits of 90% prediction intervals. Prediction intervals were calculated incorporating three different levels of variation: (1) only between-substance variation, (2) between-substance and between-study variation, and (3) between-substance, between-study, and within-study variation represented by the synthetic variable as described above. The upper prediction interval limit is commonly used in chemical risk assessment, in contrast to simply predicting the most likely value, to ensure that values for the majority of new substances fall below the upper limit and are thus not underestimated. Thus, for example in the third condition, 95% of absorption means for new substances measured in new studies using various different concentrations would be expected to be equal to or smaller than the prediction interval upper limit. Posterior distributions for the upper limits of prediction intervals were obtained by adding the square root of the total variance across random effects multiplied by the z-score for the 95th quantile to the linear predictor.

An additional source of variation, identity of the skin donor, was not accounted for as previous analyses in EFSA GD2017 concluded that inclusion of skin donor identity as a source of variation would not strongly influence the final values, and also because skin donor identity was not reliably recorded in the present dataset (many missing values and donor identifiers in original study reports were often not unique). Donor identity was also not incorporated into the final analysis deriving default values in EFSA GD2017. We expect that skin samples from a donor would mostly be used in only one study, and the variation introduced by using several samples from the same donor would thus already be captured by the study random effect.

As per EFSA GD2017, we present medians and 95th percentile values of the posterior distributions of the upper prediction interval limits. The latter measure represents the most conservative model-derived basis for determining the final default values, as it also takes into account uncertainty in the upper limit of the prediction interval. A schematic representation of the approach is shown in Figure 2B. In addition to results presented in EFSA GD2017, we show full posterior predictions for the average effects of concentration status and formulation type category keeping the corresponding other predictor at its reference category. We also report medians and 95% highest posterior density intervals (HPDIs). Model diagnostics are shown in the Supplementary Information.

### 2.3. Comparative Datasets

We compared the default values obtained in this study using the empirical approach to equivalently obtained values published in Sarti et al. 2025 [10] and EFSA GD2017 [9], and default values obtained in this study using the modelling approach to published values in EFSA GD2017. The underlying in vitro absorption data in Sarti et al. [10] and EFSA GD2017 were generated and analysed based on similar principles as the data for this publication but with some differences (see Section 2.3.1 and Section 2.3.2).

#### 2.3.1. EFSA2017

Default dermal absorption values suggested in EFSA GD2017 are based on a dataset containing data from 415 Good Laboratory Practice- and OECD-compliant in vitro human dermal absorption studies, 291 collated by the European Crop Protection Association (ECPA) and 124 collated by the BfR [6]. Studies were conducted between 1997 and 2014, evaluated according to the 2012 EFSA Guidance on dermal absorption [22], and assessed as described in [23]. The dataset contained data on 189 agrochemical active substances in 24 formulation types. A total of 14 studies, 95 substances, and 14 formulation types overlapped with the present dataset. Exposure duration ranged from 6–24 h. Concentrates were products in their commercially available concentration. Absorption was calculated as for the BfR2024 dataset, but only tape strips 1 and 2 were excluded.

#### 2.3.2. Sarti et al. 2025

The dataset consisted of a total of 759 Good Laboratory Practice- and OECD-compliant studies, 468 of which were evaluated by the ProHuma Institute of Scientific Studies in combination with the same 291 ECPA studies that were part of the EFSA 2017 dataset [6]. Studies were conducted between 2012 and 2019. The dataset contained data on 248 active substances in 25 formulation types. Sixteen formulation types overlapped with the present dataset. In contrast to EFSA GD2017 and this study, ZC and CS formulation types (see Table 1) were classified as water-based. Exposure duration ranged from 6–10 h. Concentrates were products in their commercially available concentrations. The dataset was not published, so a detailed comparison of substances or concentrations was not made. Absorption was calculated as for the BfR2024 dataset, but only tape strips 1 and 2 were excluded. The study calculated default values empirically following analysis performed in EFSA GD2017, but applied a different modelling approach (Bayesian Additive Regression Trees).

## 3. Results

### 3.1. Comparison of Default Values Based on Empirical Percentiles

**Figure 3:**
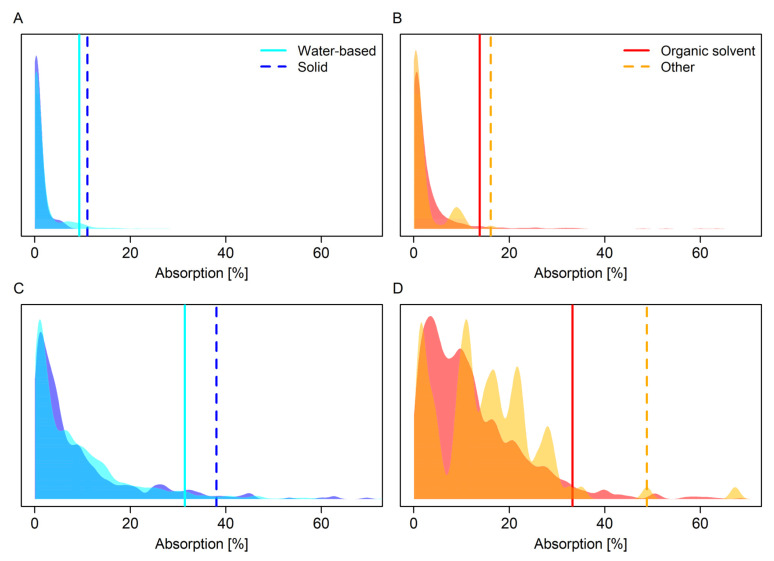
Density plots for empirical distributions of percent active substance absorbed by formulation type category and concentration status obtained in this study. (**A**,**B**) Concentrate. (**C**,**D**) Dilution. Formulation type categories are plotted in pairs to reduce overplotting. Vertical lines show upper 95% confidence limits for the 95th percentiles of the distributions. Concentrates represent the undiluted product; dilutions are intended in-use dilutions of a product. The *y*-axis shows the probability density.

Full distributions of observed absorption values for the eight different categories of concentration status and formulation type category in the BfR2024 dataset are shown in Figure 3. Default values derived from these distributions and from datasets analysed in EFSA GD2017 and Sarti et al. 2025 [10] using the same methodology are shown in Table 3. For concentrates, when values were available for comparison, values were lower than or the same as values obtained in EFSA GD2017 (absolute difference in absorption: 0–6%) and somewhat larger than values obtained in Sarti et al. [10] (absolute difference in absorption: 4–8%). For dilutions, values were considerably lower than values obtained in EFSA GD2017 (absolute difference in absorption: 12–22%) and also lower than values obtained in Sarti et al. 2025 [10] (absolute difference in absorption: 1–9%). The magnitude of values was not consistently associated with sample size (Table 3).

When concentrates were defined as target concentrations higher than 50 g/L (g/kg), the resulting empirical default values were similar to when concentrates were defined as commercially available concentrates (absolute difference in absorption: concentrate: 0–1%, dilutions: 0–3%; Table A1).

### 3.2. Model-Derived Average Effects of Formulation Type Category and Concentration Status on Absorption

**Figure 4:**
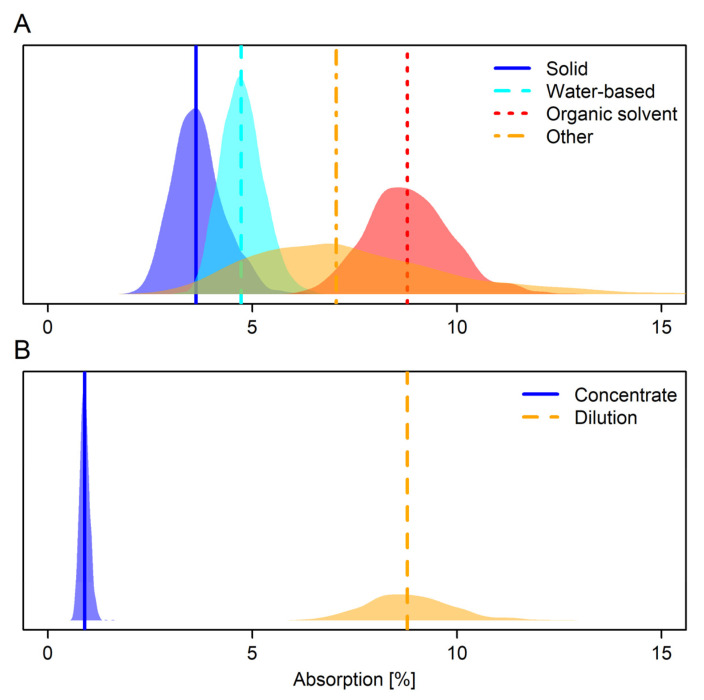
Density plot of model-derived full posterior predictions of mean dermal absorption for (**A**) formulation type category and (**B**) concentration status obtained in this study. Vertical lines show median predictions. Predictions were made for the corresponding other categorical predictor’s reference category (dilution, organic solvent). Concentrates represent the undiluted product; dilutions are intended in-use dilutions of a product. The *y*-axis shows the probability density.

Posterior model predictions indicated that there were differences in absorption among formulation type categories as well as concentrates and dilutions. Predictions were made keeping the corresponding other categorical predictor at its reference category (dilution, organic solvent). On average, active substances in solid formulations showed the lowest absorption (Median % (95% HPDI): 3.6 (2.5–4.9); Figure 4, Table 4), followed by water-based formulations (Median % (95% HPDI): 4.7 (3.8–5.8); Figure 4, Table 4). Active substances in organic solvents were most easily absorbed, and almost complete non-overlap in the posterior predictions between this category and solid and water-based formulations indicated high certainty in a difference between these formulation type categories (Median % (95% HPDI): 8.8 (6.7–11.0); Figure 4, Table 4). Substances in the ‘other’ formulation type category showed the third-highest absorption on average, but uncertainty around the median estimate was high and posterior predictions spanned almost the complete width of the posteriors of all other formulation type categories combined (Median % (95% HPDI): 7.1 (3.3–12.4); Figure 4, Table 4).

Active substances applied in dilutions were, on average, absorbed at around 10 times higher rates than active substances applied as concentrates (Median % (95% HPDI): Dilution: 8.8 (6.7–11.0); Concentrate: 0.9 (0.7–1.1); Figure 4, Table 4).

### 3.3. Comparison of Default Values Based on Modelling

**Figure 5:**
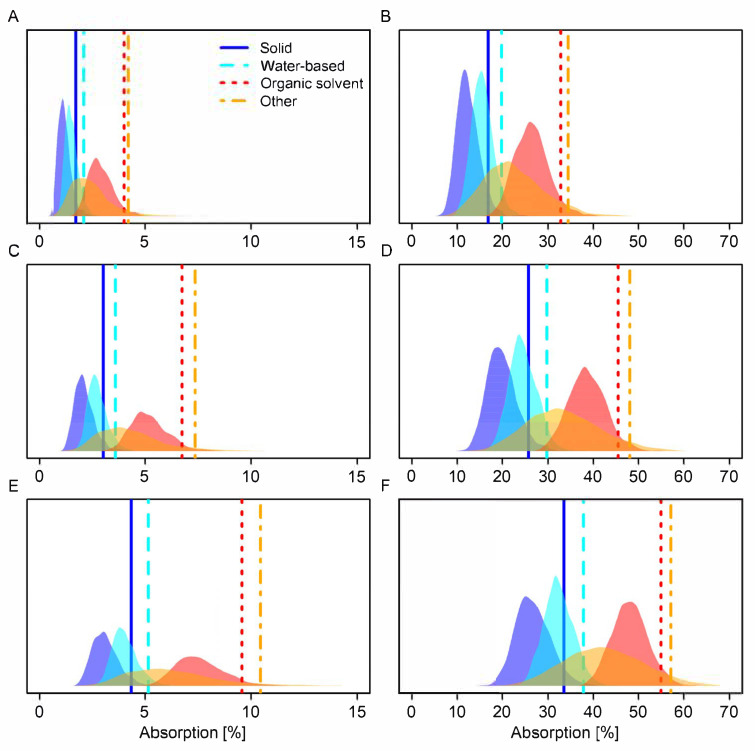
Density plots of full model-derived posterior distributions for the upper 95% credible limits of 90% prediction intervals for dermal absorption by formulation type category (solid, water-based, organic solvent, other) and concentration status (concentrate vs. dilution) obtained in this study. Vertical lines show 95th percentiles of the posterior distributions. (**A**,**C**,**E**) Concentrate. (**B**,**D**,**F**) Dilution. (**A**,**B**) Prediction interval includes only between-substance variation. (**C**,**D**) Prediction interval includes between-substance and between-study variation. (**E**,**F**) Prediction interval includes between-substance, between-study and within-study variation. Concentrates represent the undiluted product; dilutions are intended in-use dilutions of a product. The *y*-axis shows the probability density.

Table 5 and Figure 5 show model-derived default values for the datasets in EFSA GD2017 and this study based on prediction intervals incorporating three different levels of variation: (1) only between-substance variation, (2) between-substance and between-study variation, or (3) between-substance, between-study, and within-study variation. Default values are derived from prediction intervals incorporating all three sources of variation as the most conservative option.

Across all levels of variation, values obtained in this study were lower than values obtained in EFSA GD2017 (Table 5). Default values for absorption were 1–4% lower for concentrates and 1–15% lower for dilutions (Table 5). The largest absolute reduction was seen for solids applied in dilutions (absolute difference in absorption: 15%).

Relative differences in default values in comparison to values in EFSA GD2017 were considerable (relative difference in absorption concentrates: 9–50%; dilutions: 2–31%). The largest relative reduction was seen for solids applied in concentrates (relative difference in absorption: 50%). Each source of variation contributed similarly to the width of the prediction intervals (random effect variances: 0.52–0.67; Table 4, Figure 5).

When concentrates were defined as target concentrations higher than 50 g/L (or g/kg), the resulting model-derived default values were similar to when concentrates were defined as commercially available concentrates only when all three sources of variation were considered (absolute difference in absorption: 0–2%; Table 6), and considerably lower when only one or two sources of variation were considered (absolute difference in absorption: 1–11%; Table 6). This was likely due to within-study variation which was two to three times higher than the other sources of variation (within-study random effect variance: 1.20, other random effect variances: 0.31–0.56; Table A2).

## 4. Discussion

We used a new dataset of in vitro GLP- and OECD-compliant studies of dermal absorption of pesticides evaluated according to criteria defined in EFSA GD2017 to derive default values for dermal absorption for eight groups of concentration status and formulation types, empirically and by modelling. We compared the resulting values of the empirical approach to EFSA GD2017 and to Sarti et al. 2025 [10], and the resulting values for the modelling approach to EFSA GD2017 to investigate the generalisability of the currently used default values with regard to other datasets.

Empirically derived default values varied considerably among the studies—up to a difference of 22% absorption for the organic solvent category applied in dilutions—and tended to be lower for the BfR2024 and the Sarti et al. 2025 datasets, than for the EFSA GD2017 dataset. Differences among studies were not purely based on the sample size of each category. However, while calculations based on empirical distributions require the least assumptions, the generalisability of empirically derived default values to new substances is highly limited. Obtained values will be mainly driven by the specific composition of the dataset with regard to substances, formulation types, and concentrations, disregarding the fact that some substances are supported by more studies or were tested at a greater range of concentrations, as well as differences in the number of replicates. Thus, the observed differences in derived default values even among the three relatively large datasets compared here are not at all surprising. During EFSA guidance development the experts integrated the outcome of both, the empirical and the modelling approach, for setting of the default values [9]. We instead focus the discussion on the model-derived default values.

Linear mixed modelling allows estimation of prediction intervals in which a set percentage of absorption values for new substances applied in different concentrations in new studies are expected to fall. Results are less dependent on the specific composition of the underlying datasets.

Comparing model-derived values taking into account all three sources of variation, we found that default values derived with the BfR2024 dataset were also often lower than values obtained with the EFSA2017 dataset. The largest absolute difference was observed for solid formulation types applied in dilutions (15%). The differences between default values were even larger when the final grouped default values of 10% for concentrates and 50% for dilutions in water-based or solid formulations, and 25% for concentrates and 70% for dilutions in organic solvent or ‘other’ formulations were considered [9]. On an absolute scale, absorption was generally reduced more strongly for dilutions than for concentrates. On a relative scale however, reductions in default values were considerable also for concentrates; for example, the default value for solid concentrates in the BfR2024 dataset (4%; Table 5) was 50% lower than the default value for solid concentrates in the EFSA2017 dataset (8%; Table 5). Thus, even though some differences in default values on the absolute scale might seem trivial, even a small absolute reduction in the percentage of active substance absorbed could lead to a large reduction in estimated systemic exposure, particularly for high concentrations. For example, a total dose of 5000 µg of active substance applied to the skin would lead to 500 µg systemic exposure at 10%, 400 µg at 8%, and 200 µg systemic exposure at 4% absorbed substance.

As a practical consequence, the consideration of lower dermal absorption default values could, in some cases, lead to a higher number of active substance approvals or more frequent product authorisations at the national level.

The differences in model-derived default values could arise due to potential improvements in the implementation of dermal absorption studies driven by technical advancements and their strict evaluation according to the newest guidance. In this case, a revision of the default values based on more recent studies evaluated under the newest guidance might be warranted. Differences in default values due to changes in the newest guidance with regard to the assessment of in vitro studies are unlikely to be large, as the revised guidance mostly concretises pre-existing recommendations. However, the guidance has overall led to a more harmonised assessment. One concrete change is a definitive recommendation for the infrequent cases in which mass balance recovery is <95% and dermal absorption is <5%. With regard to the timing of the studies, there is evidence that the quality of in vitro dermal absorption studies has improved over time, which would favour a reconsideration of default values based on newer studies. Pieper et al. 2024 [13] found that the mass balance recovery increased for studies conducted after 2012. While improvements during this timeframe could be partly due to guidance changes, additional improvement may stem from laboratories becoming more experienced with regard to technical handling, such as the application of difficult-to-apply formulation types, standardised washing and tape stripping procedures, or the identification of receptor media that improve solubility for certain substances. About 80% of studies in the BfR2024 dataset were conducted after 2012 versus ~25% in the EFSA2017 dataset. Improvements in study implementation and assessment would not only lead to more realistic default values but also reduce the within- and between-study variation components of the prediction intervals.

Even though the modelling approach applied here increases the generalisability of the default values overall, lower values in the present dataset may also arise due to differences in the underlying datasets. Higher absorption is expected the longer the skin is exposed to the substance. Studies in BfR2024 were limited to an exposure time of 6–8 h, while 14% of studies in the EFSA2017 dataset had exposure times of 10–24 h, which could partially explain the lower default values. Additionally, formulation type categories in the EFSA2017 dataset, mainly the ‘solid’ and ‘other’ formulation type category, were represented by more formulation types than in the BfR2024 dataset, thus, possibly increasing variability within these formulation type categories, which could partially explain higher default values obtained with the EFSA2017 dataset.

When investigating the impact of using the revised definition of ‘concentrate’ and ‘dilution’ using a cut-off of 50 g/L (or g/kg) as per SCoPAFF [15], we found that when all three sources of variation where considered, model-derived default values were highly similar to values obtained when concentrates were defined as commercially available product concentrations. Thus, default values were relatively insensitive to the definition of concentrate when all three sources of variation were considered in the construction of prediction intervals. For some products, however, the alternative definition would obviously lead to drastically different systemic exposure estimates. For example, in the BfR2024 dataset, flupyradifurone was applied in a water-based formulation in a dilution at an area dose of 1160 µg/cm^2^ (116 g/L), leading to an estimated systemic exposure of 580 µg/cm^2^ when it is classified as a dilution and an estimated systemic exposure of 116 µg/cm^2^ when it is classified as a concentrate using the final default values of 10% and 50% in EFSA GD2017. However, given that at least in the BfR2024 dataset only few dilutions are redefined as concentrates using the 50 g/L cut-off, the change in systemic exposure estimates using the alternative definition is expected to be mostly protective.

When only one or two sources of variation were considered, model-derived default values tended to be considerably lower when the alternative definition was applied. This was because within-study variability explained more variation in the data than did variability between substances or studies. As a consequence, the width of prediction intervals, and therefore, suggested default values, increased more when within-study variation was considered, than when between-study variation was added to the calculation of the prediction intervals (Table 6). In contrast, when concentrates were defined as commercially available product concentrations, each added level of random effects variation added approximately equal width to the prediction interval (Table 5, Figure 5). The within-study random effect grouped replicates of the same applied concentration of the same substance in the same formulation within the same study. Given that most studies investigated only one formulation and one substance, the variation absorbed by this random effect was mostly driven by differences in applied concentrations which increased when some concentrates were now classified as dilutions and vice versa (see Figure 1). This highlights the importance of applied concentration for absorption beyond the simplistic classification into dilutions and concentrates. The dependence on concentration could be better addressed by explicitly modelling absorption as a function of concentration, or preferably applied amount of active substance per cm^2^ (dose approach) [12], which should lead to more refined, and, for most substances and concentrations, more realistic absorption values. Two relatively recent publications have applied this approach in slightly different ways to obtain predictions of absorption based on applied concentrations/amounts, while still separating data into dilutions and concentrates [12,14]. These publications specifically modelled the non-linear dependence of absorption on applied concentration/amount as a continuous variable, allowing prediction of absorption for any concentration/amount of applied substance. The dose approach has the additional advantage that it would allow an estimation of systemic exposure per exposed skin area [12].

Like these publications and others, we also found that smaller fractions were absorbed for substances applied in concentrates than substances applied in dilutions [9,10,11,12,13,14]. This counter-intuitive inversely proportional relationship is likely mainly an artifact resulting from the use of relative instead of absolute absorption. It can best be explained by a saturation effect of the skin, so that, when dermal loading is high, absolute absorption does not proportionally increase with applied dose for most substances, and thus the fraction absorbed decreases [24,25]. However, as flux across the skin is also concentration dependent, absolute absorption is usually higher for concentrates [25]. Modelling of absorption based on applied and absorbed amounts of active substance instead of percentages, taking into account the non-linearity of absorption in dependence on dose, while simultaneously allowing the relationship to vary for different substances, formulation types or co-formulants, could further refine predictions and may not require an arguably arbitrary classification as concentrates or dilutions.

In the present study, distinct peaks of the posterior distributions for absorption means of solid and water-based formulations indicated that substances in solid formulations were likely absorbed less than substances in water-based formulations indicating that separation of the two categories might be recommended (Figure 4). This general tendency was also reflected in the default values. However, absolute differences in absorption were not large (but see discussion on relative differences in absorption above), and substances in solid formulations were not consistently less absorbed than substances in water-based formulations in other studies [9,10,13] possibly making the difference a feature of this particular dataset, but see [26].

Similarly, investigation of modelled absorption means showed that, on average, absorption of substances in the ‘other’ formulation type category was less than absorption of substances in organic solvent formulations. However, mean absorption for substances in ‘other’ formulations was poorly characterised with the posterior distribution spanning the full breadth of posterior distributions for the other three formulation type categories (Figure 4). In concert with the multimodality of the raw data distribution for the ‘other’ category (Figure 3), this indicates that ‘other’ formulations are not a uniform grouping. In CS formulations, for example, the active substance is typically encased in polymer- or lipid-based shells and released gradually upon application [27]. This slower release reduces the immediate availability of the active compound on the skin’s surface, which may result in lower absorption rates compared to non-encapsulated formulations [28]. On the other hand, gel formulations (GD) often increase absorption via various different mechanisms [29]. Therefore, default values for formulation types in this category would be most strongly refined by formulation type-specific default values, or by classification into existing categories as was done for formulation types CS and ZC in [10]. Grouping of organic solvent and ‘other’ formulation type categories is justifiable with the current approach because both contain formulation types that strongly catalyse absorption, and thus produce similarly high default values for each category, but not because the categories are per se similar.

Any absorption model is only as good as the underlying data and the physiological relevance of dermal absorption assessed in in vitro studies is paramount for realistic estimates. Current in vitro testing takes into account some factors, such as skin barrier integrity or skin surface temperature, but the human skin samples used in absorption studies are obtained from surgeries, tissue banks, or other sources, are stored frozen until analysis and are thus generally considered not metabolically active. To obtain even more realistic estimates of absorption, future in vitro studies could be based on metabolically competent 3D-skin models which could also include metabolism by the skin microbiome [30,31].

## 5. Conclusions

By analysing a new dermal absorption dataset, we obtained default values that were often considerably lower than values previously obtained through modelling and also lower than final suggestions for default values made in EFSA GD2017. This suggests that a revision of the default dermal absorption values based on newer studies evaluated under the current guidance may be warranted. Further, the current approach of categorizing concentrations into dilutions and concentrates, and the grouping of formulation types into categories is a practical approach to obtaining default values that are easily implementable in risk assessment. However, given the large amount of now available data, especially considering the possibility of compilation of data across various agencies, a predictive model of absorption based on applied and absorbed amounts that takes into account the non-linearity of the absorption process, as well as formulation types, and substance-specific and potentially influential experimental variables could predict absorption with high accuracy leading to more realistic estimated systemic exposure values for new substances. Such a model could be implemented on an online platform allowing risk assessors to easily obtain values for new substances applied in any concentration.

## Figures and Tables

**Table 1:** Overview of sample size by formulation type category and concentration status.

Formulation Type Category	Dilution/ Concentrate	Replicates	Studies	Substances	Formulation Types
Organic solvent	Concentrate	847	106	68	7
Other	Concentrate	83	11	8	3
Water-based	Concentrate	1268	153	86	4
Solid	Concentrate	351	47	37	6
Organic solvent	Dilution	1490	119	66	6
Other	Dilution	103	9	7	2
Water-based	Dilution	1771	153	83	3
Solid	Dilution	427	47	33	4
Overall		6340	356	155	20

**Table 2:** Overview of formulation type categories.

Formulation Type Category	Formulation Types
Organic solvent	EC, EW, DC, ME, OD, OL, SE
Other	GD, CS, ZC
Water-based	FS, SC, SD, SL
Solid	AP, DP, GR, SG, WG, WP

EC: Emulsifiable concentrate; EW: Oil in water emulsion; DC: Dispersible concentrate: ME: Micro-emulsion; OD: Oil dispersion; OL: Oil miscible liquid; SE: Suspo-emulsion; GD: Gel for direct application; CS: Capsule suspension; ZC: A mixed formulation of CS and SC; FS: Flowable concentrate for seed treatment; SC: Suspension concentrate; SD: Suspension concentrate for direct application; SL: Soluble concentrate; AP: Other powder; DP: Dustable powder; GR: Granule; SG: Water soluble granule; WG: Water dispersible granule; WP: Wettable powder.

**Table 3:** Comparison table of *empirical* 95th percentiles of percent absorption, and upper 95% confidence limits of confidence intervals around these percentiles obtained by analyses in EFSA GD2017 [9], Sarti et al. 2025 [10] and this study. Sarti et al. 2025 [10] did not provide 95th percentile values. The upper 95% CI limits represent the most conservative estimates for empirically derived default values. Concentrates represent the undiluted product; dilutions are intended in-use dilutions of a product.

		This Study			EFSA2017			Sarti et al. 2025	
Formulation Type Category	Dilution/ Concentrate	95th Perc. ^1^	95% CI Limit ^2^	Replicates	95th Perc. ^1^	95% CI Limit ^2^	Replicates	95% CI Limit ^2^	Replicates
Organic solvent	Concentrate	11	14	847	18	20	1153	10	2150
Other	Concentrate	9	16	83	20	-	131	8	73
Water-based	Concentrate	8	9	1268	8	10	1073	4	2509
Solid	Concentrate	7	11	351	8	11	471	3	949
Organic solvent	Dilution	31	33	1490	49	55	1553	42	3318
Other	Dilution	29	49	119	56	61	105	-	8
Water-based	Dilution	30	31	1771	40	44	1567	37	3463
Solid	Dilution	33	38	427	51	57	710	39	1510

^1^ 95th percentile. ^2^ Upper 95% CI limit for the 95th percentile.

**Table 4:** Mean estimates and 95% confidence intervals for model coefficients (α, β), random effect variances (σ^2^) and the correlation between substance random effects (ρ) for the fraction of active substance absorbed. The dependent variable was logit-transformed. Concentrates represent the undiluted product; dilutions are intended in-use dilutions of a product.

Parameter	Mean	95% CI Lower Bound	95% CI Upper Bound	Effective Samples
α Intercept	−2.34	−2.63	−2.11	2000
β Concentrate	−2.37	−2.56	−2.17	2000
β Other	−0.24	−0.89	0.50	2000
β Solid	−0.94	−1.32	−0.56	2000
β Water-based	−0.67	−0.95	−0.39	2000
σ^2^ Substance:Dilution ^1^	0.64	0.38	0.90	1753
σ^2^ Substance:Concentrate ^2^	0.52	0.27	0.81	1472
σ^2^ Study ID ^3^	0.67	0.48	0.85	1650
σ^2^ Within-study ^4^	0.59	0.49	0.70	1684
ρ (σ^2^ S:D, σ^2^ S:C) ^5^	0.20	0.01	0.40	1780

^1^ Between-substance variation for dilutions; ^2^ Between-substance variation for concentrates; ^3^ Between-study variation; ^4^ Within-study variation; ^5^ Correlation between the between-substance variation for dilutions and concentrates.

**Table 5:** *Model-derived* medians and 95th percentiles of the posterior distributions for the upper credible limits of 90% prediction intervals for percent absorption. Numbers in parentheses show values obtained in EFSA GD2017 [9]. The 95th percentile values incorporating all three sources of variation represent the most conservative estimates for model-derived default values. Concentrates represent the undiluted product; dilutions are intended in-use dilutions of a product.

Formulation Type Category	Dilution/ Concentrate	Posterior Median ^1^	95th Percentile ^1^	Posterior Median ^2^	95th Percentile ^2^	Posterior Median ^3^	95th Percentile ^3^
Organic solvent	Concentrate	3 (5)	4 (7)	5 (8)	7 (10)	7 (11)	10 (14)
Other	Concentrate	2 (3)	4 (6)	4 (5)	7 (8)	6 (7)	10 (11)
Water-based	Concentrate	1 (3)	2 (4)	3 (4)	4 (6)	4 (6)	5 (8)
Solid	Concentrate	1 (3)	2 (4)	2 (4)	3 (6)	3 (6)	4 (8)
Organic solvent	Dilution	26 (37)	33 (45)	38 (48)	45 (56)	48 (57)	55 (64)
Other	Dilution	22 (27)	34 (39)	33 (37)	48 (49)	42 (46)	57 (58)
Water-based	Dilution	15 (24)	20 (30)	24 (33)	30 (39)	32 (41)	38 (47)
Solid	Dilution	12 (24)	17 (30)	20 (33)	26 (39)	26 (41)	33 (48)

^1^ Prediction interval includes only between-substance variation. ^2^ Prediction interval includes between-substance and between-study variation. ^3^ Prediction interval includes between-substance, between-study, and within-study variation.

**Table 6:** *Model-derived* medians and 95th percentiles of the posterior distributions for the upper credible limits of 90% prediction intervals for percent absorption. The 95th percentile values incorporating all three sources of variation represent the most conservative estimates for model-derived default values. Concentrates were defined as concentrations higher than 50 g/L (g/kg). Numbers in parentheses show values obtained when concentrates were defined as commercially available product concentrations.

Formulation Type Category	Dilution/ Concentrate	Posterior Median ^1^	95th Percentile ^1^	Posterior Median ^2^	95th Percentile ^2^	Posterior Median ^3^	95th Percentile ^3^
Organic solvent	Concentrate	2 (3)	2 (4)	3 (5)	4 (7)	7 (7)	9 (10)
Other	Concentrate	1 (2)	2 (4)	2 (4)	4 (7)	5 (7)	9 (10)
Water-based	Concentrate	1 (1)	1 (2)	2 (3)	2 (4)	4 (4)	5 (5)
Solid	Concentrate	1 (1)	1 (2)	1 (2)	2 (3)	3 (3)	4 (4)
Organic solvent	Dilution	20 (26)	26 (33)	28 (38)	35 (45)	46 (48)	53 (55)
Other	Dilution	16 (22)	27 (34)	23 (33)	37 (48)	40 (42)	55 (57)
Water-based	Dilution	13 (25)	17 (20)	19 (24)	24 (30)	34 (32)	40 (38)
Solid	Dilution	9 (12)	13 (17)	14 (20)	19 (26)	26 (26)	33 (33)

^1^ Prediction interval includes only between-substance variation. ^2^ Prediction interval includes between-substance and between-study variation. ^3^ Prediction interval includes between-substance, between-study, and within-study variation.

## Data Availability

The data and R-script necessary to replicate the analyses presented in this manuscript are published as Supplementary Data.

## Supplementary Materials

The following supporting information can be downloaded at https://www.mdpi.com/article/10.3390/toxics13110925/s1, File S1: Toxics_dataset.xlsx, File S2: Toxics_R script.r, File S3: Toxics_SI.docx (References [32,33] are cited in the Supplementary Materials).

## Abbreviations

The following abbreviations are used in this manuscript:

PPP	Plant protection product
EFSA	European Food Safety Authority
GLP	Good Laboratory Practice
BfR	German Federal Institute for Risk Assessment
SCoPAFF	Standing Committee on Plants, Animals, Food and Feed
OECD	Organization for Economic Co-operation and Development
ECPA	European Crop Protection Association
HPDI	Highest posterior density interval

## Appendix A

**Table A1:** Table of *empirical* 95th percentiles of percent absorption, and upper 95% confidence limits of confidence intervals around these percentiles. The upper 95% CI limit represents the most conservative estimate for empirically derived default values. Concentrates were defined as concentrations higher than 50 g/L (g/kg).

Formulation Type Category	Dilution/ Concentrate	95th Perc. ^1^	95% CI Limit ^2^	Replicates
Organic solvent	Concentrate	11 (11)	15 (14)	595
Other	Concentrate	10 (9)	16 (16)	67
Water-based	Concentrate	8 (8)	9 (9)	1143
Solid	Concentrate	6 (7)	11 (11)	273
Organic solvent	Dilution	30 (31)	32 (32)	1742
Other	Dilution	29 (29)	49 (49)	119
Water-based	Dilution	29 (30)	31 (31)	1896
Solid	Dilution	31 (33)	35 (38)	505

^1^ 95th percentile. ^2^ Upper 95% CI limit for the 95th percentile.

**Table A2:** Mean estimates and 95% confidence intervals for model coefficients (α, β), random effect variances (σ^2^) and the correlation between substance random effects (ρ) for the fraction of active substance absorbed. The dependent variable was logit transformed. Concentrates were defined as concentrations higher than 50 g/L (g/kg).

Parameter	Mean	95% CI Lower Bound	95% CI Upper Bound	Effective Samples
α Intercept	−2.61	−2.88	−2.36	2134
β Concentrate	−2.33	−2.53	−2.12	2000
β Other	−0.25	−0.99	0.48	2000
β Solid	−0.92	−1.34	−0.50	2000
β Water-based	−0.53	−0.84	−0.27	2000
σ^2^ Substance:Dilution ^1^	0.56	0.32	0.82	1687
σ^2^ Substance:Concentrate ^2^	0.31	0.12	0.53	779
σ^2^ Study ID ^3^	0.49	0.28	0.71	986
σ^2^ Within-study ^4^	1.20	1.04	1.39	1310
ρ (σ^2^ S:D, σ^2^ S:C) ^5^	0.31	0.12	0.53	898

^1^ Between-substance variation for dilutions; ^2^ Between-substance variation for concentrates; ^3^ Between-study variation; ^4^ Within-study variation; ^5^ Correlation between the between-substance variation for dilutions and concentrates.
