# Supplementary material for: Variability Between Datasets and Statistical Approaches—Rethinking Estimation of Default Dermal Absorption Values for Risk Assessment
Source: Toxics. 2025 Oct 29;13(11):925. doi: 10.3390/toxics13110925 (PMC12656691; doi:10.3390/toxics13110925)
Supplement: Supplementary file 1 [file toxics-13-00925-s001.zip › File S3. Toxics_SI-english done -proof.docx]

Supporting Information

**Toxics**

Variability between datasets and statistical approaches - rethinking estimation of default dermal absorption values for risk assessment

Veronika Städele, Sabine Martin and Korinna Wend

Model diagnostics

By default, *MCMCglmm* only runs a single chain; storing multiple chains in the same model object is not possible. To assess model convergence, we ran the model three more times. We conducted a posterior predictive check by simulating 50 datasets based on the first fitted model and visualized the comparison between the simulated and observed data in Figure S1. We assessed convergence among the four chains by calculating the R-hat [1] diagnostic implemented in the *posterior R* package [2]. R-hat was smaller than 1.001 for all parameters. Trace and density plots showing chains and posterior distributions for each parameter are shown in Figure S2. Note that these diagnostics were not presented in EFSA GD2017 [3].


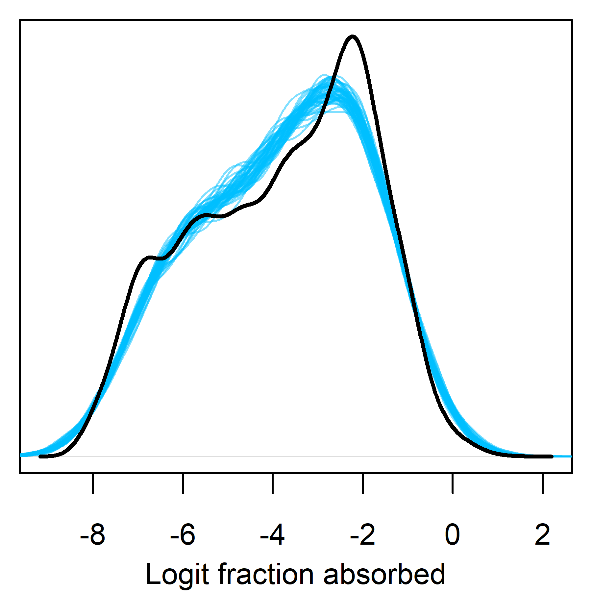


**Figure S1** Density plot comparing 50 simulated datasets (blue lines) with the observed distribution (black line). The y-axis shows the probability density.


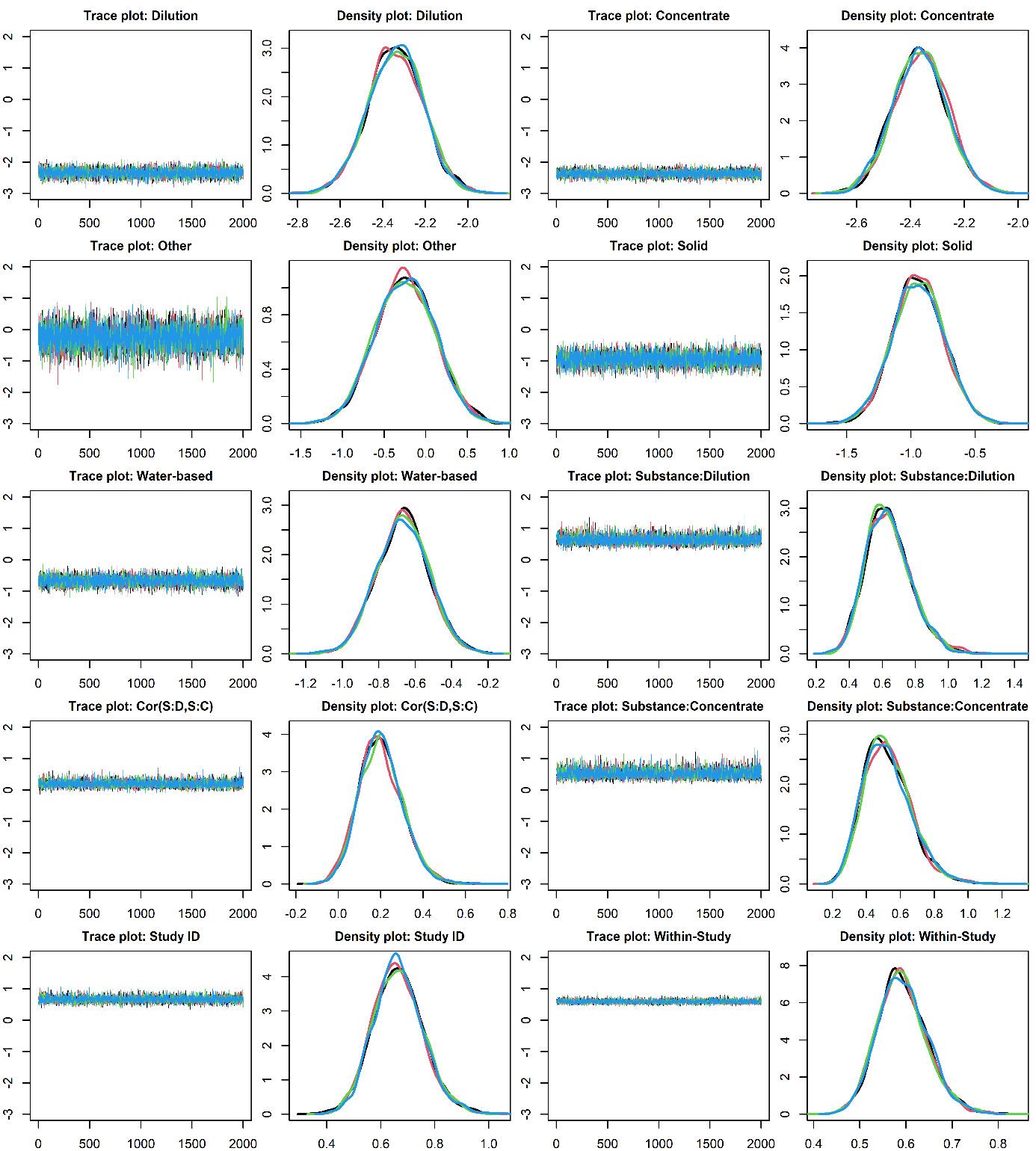


**Figure S2** Trace and density plots for four chains represented by four different colors. Trace plots show parameter values (y-axis) across 2000 iterations (x-axis). Density plots show the probability density (y-axis) across 2000 parameter values (x-axis). Parameters are the same as described for Table 4 in the main manuscript.

References

1. Vehtari, A.; Gelman, A.; Simpson, D.; Carpenter, B.; Bürkner, P.-C. Rank-normalization, folding, and localization: An improved R-hat for assessing convergence of MCMC (with Discussion). *Bayesian Analysis* **2021**, *16*, 667-718, doi:10.1214/20-BA1221.

2. Bürkner, P.; Gabry, J.; Kay, M.; Vehtari, A. *posterior: Tools for Working with Posterior Distributions*, R package version 1.6.1; 2025.

3. EFSA; Buist, H.; Craig, P.; Dewhurst, I.; Hougaard Bennekou, S.; Kneuer, C.; Machera, K.; Pieper, C.; Marques, D.C.; Guillot, G., et al. Guidance on dermal absorption. *EFSA J* **2017**, *15*, e04873, doi:10.2903/j.efsa.2017.4873.
